# Inflammatory Bowel Disease and Pregnancy: Is It a Marker for Adverse Outcomes?

**DOI:** 10.1055/s-0042-1756149

**Published:** 2022-11-29

**Authors:** Rita Vicente Costa, Carolina Simões, Luís Correia, Luísa Pinto

**Affiliations:** 1 Gynecology and Obstetris Service, Hospital Distrital de Santarém, Santarém, Portugal; 2 Gastroenterology Service, Hospital de Santa Maria, Lisboa, Portugal; 3 Gynecology and Obstetrics Service, Hospital de Santa Maria, Lisboa, Portugal

**Keywords:** inflammatory bowel diseases, pregnancy, retrospective studies, doenças inflamatórias intestinais, gravidez, estudos retrospectivos

## Abstract

**Objective** To assess obstetric/puerperal/neonatal outcomes in an inflammatory bowel disease (IBD) population and to analyze disease characteristics that may be associated to adverse outcomes.

**Methods** Retrospective descriptive analysis including 47 pregnant wom e n with IBD (28 with Crohn's disease – CD and 19 with ulcerative colitis – UC) who delivered between March 2012 and July 2018 in a tertiary hospital. We reviewed clinical records to extract demographic information, previous medical history, disease subtype, activity, severity, treatment, and obstetric, puerperal, and neonatal outcome measures.

**Results** Obstetric and neonatal complications (composite outcomes) occurred in 55.3% and 14.6% of the IBD population, respectively, and were more frequent in UC patients. Preterm birth (PTB), preeclampsia, anemia, low birth weight (LBW), and neonatal death were also more frequent in UC patients. The rate of postpartum hemorrhage (PPH) was 14.9%, and it was higher in CD patients. Women with active IBD had more obstetric/neonatal adverse outcomes (fetal growth restriction and LBW in particular) and cesarean sections. Patients with medicated IBD had less obstetric/neonatal complications (PTB and LBW in specific) and cesarean sections but more PPH.

**Conclusion** Women with IBD may have an increased risk of obstetric/puerperal/neonatal adverse outcomes. Ulcerative colitis patients had more obstetric and neonatal complications, whereas PPH was more frequent if CD patients. Other disease characteristics were considered, which allowed a better understanding of their possible influence. Although more research is needed, this work reinforces the importance of adequate surveillance to allow prompt recognition and treatment of complications.

## Resumo

**Objetivo** Avaliar os desfechos obstétricos/puerperais/neonatais em uma população com doença inflamatória intestinal (DII) e analisar as características da doença, que podem estar associadas a desfechos adversos.

**Métodos** Análise descritiva retrospectiva incluindo 47 gestantes com DII (28 com doença de Crohn – DC e 19 com retocolite ulcerativa – RCU) que deram à luz entre março de 2012 e julho de 2018 em um hospital terciário. Revisamos os registros clínicos para extrair informações demográficas, histórico médico prévio, subtipo da doença, atividade, gravidade, tratamento e medidas de resultados obstétricos, puerperais e neonatais.

**Resultados** As complicações obstétricas e neonatais (desfechos compostos) ocorreram em 55,3% e 14,6% da população com DII, respectivamente; e foram mais frequentes em pacientes com RCU. Nascimento prematuro (PTB), pré-eclâmpsia, anemia, baixo peso ao nascer (BPN) e óbito neonatal também foram mais frequentes em pacientes com RCU. A taxa de hemorragia pós-parto (HPP) foi de 14,9% e foi maior em pacientes com DC. Mulheres com DII ativa tiveram mais desfechos obstétricos/neonatais adversos (restrição de crescimento fetal e BPN em particular) e cesarianas. Pacientes com DII medicada tiveram menos complicações obstétricas/neonatais (PTB e BPN em específico) e cesarianas, mas mais HPP.

**Conclusão** Mulheres com DII podem ter um risco aumentado de desfechos adversos obstétricos/puerperais/neonatais. As pacientes com RCU apresentaram mais complicações obstétricas e neonatais, enquanto a HPP foi mais frequente em pacientes com DC. Outras características da doença foram consideradas, o que permitiu uma melhor compreensão de sua possível influência. Embora mais pesquisas sejam necessárias, este trabalho reforça a importância de uma vigilância adequada para permitir o reconhecimento e o tratamento imediatos das complicações.

## Introduction

Inflammatory bowel disease (IBD) is characterized by a chronic non-infectious inflammation of the gastrointestinal tract, and it includes Crohn's disease (CD), ulcerative colitis (UC), and indeterminate colitis. 1 The prevalence in Portugal is around 40 to 60 per 100,000. 1 The peak incidence coincides with reproductive age. 2 Inflammatory bowel disease can reduce fertility, especially if the disease is active and is associated with an increased risk of obstetric complications due to malnutrition, inflammation, and medication. 2 3

Some studies indicate that IBD may contribute to the increased incidence of preterm birth (PTB), low birth weight (LBW), and antepartum hemorrhage, but the conclusions of the articles are not unanimous. 4 5 6 7 8 9 Although there is also some controversy, most publications report that IBD does not confer a greater risk of congenital anomalies. The incidence of preeclampsia does not seem to be affected by IBD. 10 Adverse obstetric outcomes are more frequent if the disease is active in the periconceptional period or if there are exacerbations during pregnancy, so the risk of discontinuing the medication may be greater than the possible adverse effects. 8 9 10 11 12 13 14 15 16 17 18 19 20 21 22 23 24 25 However, some of the drugs used in the treatment of IBD can affect fertility or induce pregnancy complications and should be replaced by other alternatives when planning a future pregnancy. Cesarean delivery tends to be more frequent in women with IBD and is indicated in cases of active perianal disease. 4 26 In other cases of IBD, the type of delivery should be dictated by obstetric criteria.

This study analyses the potential effect of IBD in obstetric, puerperal, and perinatal outcomes, reinforcing the importance of adequate gastroenterological and obstetric surveillance. The type of IBD (CD or UC), disease activity, extent, treatment, and other IBD characteristics were also described, which provides additional information that most previous studies did not include.

## Methods

We carried out a retrospective descriptive analysis study in which we included 47 pregnant women with IBD (28 with CD and 19 with UC) followed at the Maternal-Fetal Medicine Department at Santa Maria Hospital, who delivered between March 2012 and July 2018. We described obstetric, puerperal, and neonatal adverse outcomes in our IBD sample in its totality but also in addressing several disease particularities (subtype, activity, severity, duration, and treatment).

We reviewed medical records from gastrenterology and obstetric routine appointments and urgency room visits to extract the demographic information, previous medical history, disease type/activity/severity/therapy, and outcome measures related to pregnancy, delivery, puerperium, and newborn. The software used for statistical analysis was the IBM SPSS Statistics for Windows version 23.0 (IBM Corp, Armonk, NY, USA). This study was approved by the institutional review board.

Obstetric complications included PTB (birth before 37 weeks of gestation), vaginal bleeding in the 3 ^rd^ trimester, fetal growth restriction (FGR), spontaneous and therapeutic abortion, stillbirth, placental abruption (placental detachment from the uterus before delivery), premature rupture of membranes (which, by definition, occurs before labour), (PROM)—at term and preterm—prolonged PROM (if >18 hours prior to delivery), chorioamnionitis, preeclampsia, eclampsia, congenital anomalies, intrahepatic cholestasis of pregnancy, anemia, oligohydramnios, short cervix, and gestational diabetes.

Abortion was defined as a nonviable intrauterine pregnancy up to 20 weeks of gestation (spontaneous if without intervention, or therapeutic if it was a purposed termination for medical reasons). Stillbirth refers to delivery of a fetus ≥ 20 weeks of gestation with no signs of life. Fetal growth restriction was defined as estimated fetal weight below 3 ^rd^ centile or below 10 ^th^ centile when associated to fluxometric changes. Congenital anomalies comprise a wide range of abnormalities of body structure or function that are present at birth and are of prenatal origin. Preeclampsia refers to the new onset of hypertension (systolic/diastolic blood pressure ≥ 140 mmHg/90 mmHg in 2 occasions 4 hours apart and proteinuria or the new onset of hypertension and significant end-organ dysfunction with or without proteinuria after 20 weeks of gestation or postpartum in a previously normotensive woman). Eclampsia refers to the occurrence of new-onset, generalized, tonic-clonic seizures or coma in a patient with preeclampsia or gestational hypertension. Intrahepatic cholestasis of pregnancy was diagnosed when there was presence of pruritus associated with elevated total serum bile acid levels, elevated aminotransferases or both, and the absence of diseases that may produce similar laboratory findings and symptoms. Anemia during pregnancy was defined by hemoglobin < 11 g/dL in the first or third trimester or < 10.5 g/dl in the second trimester. The diagnosis of oligohydramnios and short cervix was made either by routine ultrasound or ultrasound in the emergency room. Oligohydramnios was diagnosed by obstetric ultrasound when single deepest pocket < 2 cm or amniotic fluid index ≤ 5 cm. Short cervix was detected when transvaginal ultrasound cervical length was ≤ 25 mm before 24 weeks of gestation. Gestational diabetes was diagnosed by the results of routine blood tests (either if fast glucose levels ≥ 92 mg/dl in the first trimester or between 24 ^th^ and 28 ^th^ weeks of gestation) or if glucose levels ≥ 180 mg/dl or ≥ 153 mg/dl 1 or 2 hours after ingesting 75 g of glucose, respectively (between 24 ^th^ and 28 ^th^ weeks of gestation). Chorioamnionitis was defined as maternal fever (axilary temperature ≥ 38°C or tympanic temperature ≥ 38.5°C) associated with at least 2 of the following: maternal tachycardia (> 100 bpm)/fetal tachycardia (> 160 bpm), painful uterine palpation, purulent vaginal discharge/amniotic fluid with fetid odor or leucocitosys (> 15 × 10 ^^9^ /L)/reactive protein-C elevation (> 10 mg/L).

Fetal growth restriction, oligohydramnios, and short cervix were consistently investigated in routine ultrasounds as well as anemia and gestational diabetes, in routine blood tests. We revised clinical files from routine appointments and emergency room visits to verify the presence or absence of the remaining obstetric adverse outcome.

Postpartum hemorrhage (PPH) was evaluated as the perception of ≥ 500 ml of blood after a vaginal birth or ≥ 1,000 ml of blood after a cesarean delivery.

Neonatal adverse outcomes, as a composite outcome, included: LBW (birth weight < 2,500 g), Apgar index < 7 (in the 1 ^st^ , 5 ^th^ or 10 ^th^ minute after birth), neonatal death (death in the first 28 days of life), and neonatal intensive care unit admission.

## Results

Forty-seven pregnant women with IBD were included (28 with CD and 19 with UC). Their mean age was 33.7 ± 3.7 years. The sample included 25 nulliparous and 22 multiparous patients as well as 45 single-born and 2 twin pregnancies. Some of the women had other relevant diseases or history (4 smokers, 4 with asthma, 1 with previous conization, 1 with septate uterus, 1 with previous uterine perforation, 5 with obesity, 3 with chronic anemia and 1 with chronic gastritis and osteoporosis).

Extraintestinal manifestations were reported in 30.8% (n = 12) of the women (7.7%, n = 3 musculoskeletal, 7.7%, n = 3 dermatological/oral and 7.7%, n = 3 hematological, 5.1%, n = 2 hepatobiliary and 2.6%, n = 1 with > 1 extraintestinal manifestation).

We collected the necessary data to characterize IBD with the Montreal classification in the 18 cases for which this information was available. Regarding the location of CD, the terminal ileon and the ileon and colon were the most affected areas (37.5%, n = 6 each), followed by the colon alone (25%, n = 4). We also evaluated the behavior of CD. In 43.8% (n = 7) of the cases, the disease was non-stenosing and non-penetrating; in 25% (n = 4), it was strict; in 12.5% (n = 2), it was penetrating; and in 18.8%, (n = 3) a combination of the above was described. As for the extension of the UC, it was characterized only in 2 cases: 1 with left colitis and 1 with pancolitis. Active perianal disease was documented in 29.4% of cases (n = 10), which constituted an indication for elective cesarean section. The age at diagnosis was ≤ 16 years in 5.6% (n = 1) and 17 to 40 years in 94.4% (n = 17) of cases. The average time since the diagnosis of IBD was 7.61 years. The diagnosis of IBD had been made ≤ 5 years ago in 41.7% (n = 15) of pregnant women, > 5 years ago in 58.3% (n = 21), and > 10 years ago in 36.1% (n = 13).

During the periconceptional period, according to the endoscopic Mayo classification, 60% (n = 3) of pregnant women with UC had a score 3, and 40% (n = 2) had a score 2. Among those with CD, the majority (87%; n = 20) was classified as being in remission, while 13% (n = 3) had mild disease (Harvey-Bradshaw clinical classification).

Most pregnant women (85.7%; n = 36) with IBD were medicated for the pathology, and 26.2% (n = 11) were medicated with more than 1 drug. Salicylates was the most used class of drugs (47.6%; n = 20), followed by thiopurines (38.1%; n = 16), biologicals (23.8%; n = 10 - discontinued on average at 23 weeks of pregnancy), corticosteroids (4.76%; n = 2), and antibiotics (2.4%; n = 1). Almost half of those with IBD (45.2%; n = 19) were medicated with immunosuppressants. Among pregnant women with IBD, 25.5% (n = 12) had previously undergone intestinal surgery due to CD.

Almost a third (31.4%; n = 11) of pregnant women with IBD had active disease in the periconceptional period, of which 81.8% (n = 9) remained active during pregnancy. Overall, 41.7% (n = 15) of pregnant women manifested IBD activity during pregnancy. Among pregnant women with inactive disease in the periconceptional period, 25% (n = 6) had exacerbation of the disease during pregnancy. Only 9.1% (n = 3) of pregnant women had active disease in the puerperium and, in all of these cases, IBD activity had already been documented during pregnancy.

Obstetric complications, evaluated as a composite outcome (PTB, vaginal bleeding in the 3 ^rd^ trimester, FGR, placental abruption, PROM at term and preterm, prolonged PROM, spontaneous and therapeutic abortions, stillbirths, congenital anomalies, chorioamnionitis, preeclampsia, eclampsia, intrahepatic cholestasis of pregnancy, anemia, oligohydramnios, short cervix and gestational diabetes) occurred in 55.3% (n = 26) of pregnant women with IBD, and women with UC had a higher rate of obstetric adverse outcomes than those with CD (68.4%, n = 13 vs 46.4%, n = 13) ( Table 1 ).

**Table 1:** Obstetric complications

	IBD (n = 47)	CD (n = 28)	UC (n = 19)
PROM* at term	19.1% (9)	21.4% (6)	15.8% (3)
Preterm birth	8.5% (4)	7.1% (2)	10.5% (2)
Fetal growth restriction	10.6% (5)	10.7% (3)	10.5% (2)
Preterm PROM*	2.1% (1)	3.6% (1)	0% (0)
Placental abruption	2.1% (1)	0% (0)	5.3% (1)
Preeclampsia	4.3% (2)	3.6% (1)	5.3% (1)
Intrahepatic cholestasis of pregnancy	8.5% (4)	7.1% (2)	7.1% (2)
Anemia	4.3% (2)	0% (0)	7.1% (2)
Vaginal bleeding in the third trimester	4.3% (2)	3.6% (1)	5.3% (1)
Oligohydramnios	2.1% (1)	3.6% (1)	0% (0)
Short cervix	6.4% (3)	7.1% (2)	5.3% (1)
Gestational diabetes	8.5% (4)	14.3% (4)	0% (0)
Therapeutic abortion (selective feticide)	2.1% (1)	3.6% (1)	0% (0)

Abbreviations: CD, Crohn's disease; IBD, inflammatory bowel disease; PROM, premature rupture of membranes; UC, ulcerative colitis.

After a joint decision with the parents, 1 selective feticide was performed at 18 weeks (by ultrasound-guided umbilical cord occlusion) due to severe FGR in a monochorionic twin pregnancy, so there were only 48 live births. There were no spontaneous abortions, stilllbirth, cases of eclampsia, chorioamnionitis, prolonged rupture of membranes, or congenital anomalies.

Preterm and term PROM, FGR, oligohydramnios, short cervix, gestational diabetes, and feticide were more frequent in CD than in UC ( Table 1 ). Preterm birth, placental abruption, preeclampsia, anemia, and vaginal bleeding in the third trimester were more prevalent in pregnant women with UC than in those with DC. The same prevalence was observed in the DC and CU groups regarding intrahepatic cholestasis of pregnancy (7.1%) ( Table 1 ).

Operative delivery, which included vaccum and forceps deliveries as well as all cesarean sections, was more frequent in pregnant women with CD (78.6% in CD vs 65% in UC) ( Table 2 ).

**Table 2:** Route of delivery

Route of delivery	IBD (n = 48)	CD (n = 28)	UC (n = 20)
**Operative delivery**	72.9% (35)	78.6% (22)	65% (13)
**Cesarean section**	39.6% (19)	50% (14)	25% (5)
Elective	68.4% (13)	85.7% (12)	20% (1)
Urgent/emergent	31.6% (6)	14.3% (2)	80% (4)
**Forceps delivery**	6.2% (3)	10.7% (3)	0% (0)
**Vaccum delivery**	27.1% (13)	17.9% (5)	40% (8)
**Eutocic delivery**	27.1% (13)	21.4% (6)	35% (7)

Abbreviations: CD, Crohn's disease; IBD, inflammatory bowel disease; UC, ulcerative colitis.

*Operative delivery included vaccum delivery, forceps delivery and all cesarean sections

There were 13 elective cesarean sections: 10 due to active perianal disease, 1 due to a previous uterine perforation, 1 due to pelvic fetal presentation and 1 due to 2 previous cesarean sections. Within the remaining cesarean sections, 1 was emergent due to placental abruption and 5 were urgent (2 due to labour dystocia and 3 to failed induction of labour). Prolonged second stage of labour was the indication for 3 forceps and 11 vaccum deliveries. The remaining 2 vaccum deliveries were motivated by fetal bradicardia in the 2 ^nd^ stage of labour.

Considering IBD characteristics, obstetric complications (composite outcome) were more frequent in women with active IBD in the periconceptional period (63.6% vs 45.8%), IBD duration > 5 years (52.4% vs 46.7%), or active perianal disease (60% vs 45.8%), but not with more severe IBD or with activity during pregnancy ( Table 3 ). Specifically regarding PTB, it was more frequent if IBD duration > 5 years (14.3% vs 6.7% if ≤ 5 years), more severe UC (33.3% if score Mayo 3 vs 0% if score 2), or active perianal disease (10% vs 8.3%) ( Table 3 ). Fetal growth restriction had a higher occurrence in women with active IBD in the periconceptional period (but not during pregnancy), more recent IBD diagnosis, or active perianal disease ( Table 3 ). We found a higher frequency of operative delivery in cases of active IBD in the periconceptional period (100%, n = 11 vs 66.7%, n = 16 if IBD in remission) and pregnancy (80% vs 76.2%), fewer time since IBD diagnosis (80% if ≤ 5 years vs 76.2% if > 5 years), more severe CD (100% if mild disease vs 80% if disease in remission), or active perianal disease (100% vs 75%) ( Table 3 ). We also verified more cesarean sections in pregnant women with active IBD in the periconceptional period (63.6%, n = 7 vs 29.2%, n = 7 if IBD in remission), diagnosis of IBD > 5 years ago (52.4%, n = 11 vs 20%, n = 3 in those with diagnosis ≤ 5 years ago), more severe IBD (0% if UC with Mayo score 2 vs 66.7% if Mayo score 3 and 45% if CD in remission vs 100% if mild disease), and also those with active perianal disease (100%, n = 10 vs 25%, n = 6) ( Table 3 ). Neonatal adverse outcomes (composite outcome) were present in 14.6% (n = 7) of the newborns of mothers with IBD, higher if UC patients (20%, n = 4 vs 10.7%, n = 3 if CD). These adverse outcomes were more frequent in cases of active IBD in the periconceptional period and during regnancy (27.3% vs 12.5% and 20% vs 14.3%, respectively), more severe CD (33.3% if mild disease vs 5% CD in remission), and also in cases of IBD diagnosis > 5 years ago (19% vs 13.3% if diagnosis ≤ 5 years), but not in cases of active perianal disease ( Table 3 ). Low birth weight was also more frequent if the disease was active (27.3%, n = 3 vs 4.2%, n = 1 in the periconceptional period and 20%, n = 3 vs 4.8%, n = 1 during pregnancy), diagnosis made > 5 years ago (14.3%, n = 3 vs 6.7%, n = 1 if diagnosis ≤ 5 years ago), and in active perianal disease cases (10%, n = 1 vs 8.3%, n = 2) ( Table 3 ). The rate of PPH in the IBD sample was 14.9% (n = 7), higher in CD patients (17.9%, n = 5) than in women with UC (10.5%, n = 2). The occurrence of PPH was higher in women with more years since the IBD diagnosis (13.3%, n = 2 if ≤ 5 years vs 19%, n = 4 if > 5 years) and more severe CD (33.3%, n = 1 if mild disease vs 15%, n = 3 if CD in remission), but not with active IBD (during the periconceptional period or pregnancy) or perianal disease ( Table 3 ).

**Table 3:** Inflammatory bowel disease characteristics and obstetric/neonatal/puerperal adverse outcomes

	Obstetric adverse outcomes (composite outcome) ^a)^	Preterm birth	Fetal growth restriction	Operative delivery ^b)^	Cesarean section	Neonatal adverse outcomes (composite outcome) ^c)^	Low birth weight	Postpartum hemorrhage
**IBD activity (periconceptional period)**								
Remission (n = 24)	45.8% (11)	12.5% (3)	4.2% (1)	66.7% (16)	29.2% (7)	12.5% (3)	4.2% (1)	20.8% (5)
Active (n = 11)	63.6% (7)	9.1% (1)	18.2% (2)	100% (11)	63.6% (7)	27.3% (3)	27.3% (3)	9.1% (1)
**IBD activity (pregnancy)**								
Remission (n = 21)	52.4% (11)	14.3% (3)	9.5% (2)	76.2% (16)	45.2% (9)	14.3% (3)	4.8% (1)	23.8% (5)
Active (n = 15)	40% (6)	6.7% (1)	6.1% (1)	80% (12)	33.3% (5)	20% (3)	20% (3)	6.7% (1)
**IBD duration**								
≤ 5 years (n = 15)	46.7% (7)	6.7% (1)	13.3% (2)	80% (12)	20% (3)	13.3% (2)	6.7% (1)	13.3% (2)
> 5 years (n = 21)	52.4% (11)	14.3% (3)	9.5% (2)	76.2% (16)	52.4% (11)	19% (4)	14.3% (3)	19% (4)
** CD severity (Harvey-Bradshaw classification) ^d)^**								
Remission (n = 20)	45% (9)	10% (2)	10% (2)	80% (16)	45% (9)	5% (1)	0% (0)	15% (3)
Mild disease (n = 3)	33.3% (1)	0% (0)	0% (0)	100% (3)	100% (3)	33.3% (1)	33.3% (1)	33.3% (1)
** UC severity (Mayo classification) ^e)^**								
Score 2 (n = 2)	100% (2)	0% (0)	50% (1)	100% (2)	0% (0)	50% (1)	50% (1)	0% (0)
Score 3 (n = 3)	66.7% (2)	33.3% (1)	0% (0)	100% (3)	66.7% (2)	33.3% (1)	33.3% (1)	0% (0)
**Perianal disease**								
Absent/inactive (n = 24)	45.8% (11)	8.3% (2)	8.3% (2)	75% (18)	25% (6)	16.7% (4)	8.3% (2)	20.8% (5)
Active (n = 10)	60% (6)	10% (1)	20% (2)	100% (10)	100% (10)	10% (1)	10% (1)	20% (2)

Abbreviations: CD, Crohn's disease; IBD, inflammatory bowel disease; UC, ulcerative colitis.

a) Obstetric adverse outcomes (composite outcome) included preterm birth, vaginal bleeding in the 3 ^rd^ trimester, fetal growth restriction, spontaneous and therapeutic abortion, stillbirth, placental abruption, premature rupture of membranes at term and preterm, prolonged premature rupture of membranes, preeclampsia, eclampsia, chorioamnionitis, congenital anomalies, intrahepatic cholestasis of pregnancy, anemia, oligohydramnios, short cervix, gestational diabetes. b) Operative delivery included vaccum delivery, forceps delivery and all cesarean sections. c) Neonatal adverse outcomes (composite outcome) included low birth weight, Apgar index < 7, neonatal death and neonatal intensive care unit admission. d) Harvey-Bradshaw classification includes 5 variables (general well-being, abdominal pain, number of liquid dejections per day, abdominal mass and complications), each with an individual score. Total score: < 5 (remission), 5–7 (mild disease), 8–16 (moderate disease) or > 16 (severe disease). e) Mayo endoscopic classification – score 0 (absence of disease or inactive disease – absence of endoscopic alterations), 1 (mild disease – erythema, decreased vascular pattern, mild friability), 2 (moderate disease – marked erythema, absent vascular pattern, friability, erosions) or 3 (severe disease – spontaneous bleeding, ulceration)

Women with medicated IBD had less obstetric and neonatal complications (composite outcomes; including PTB and LBW in specific), operative delivery, and cesarean sections but more PPH ( Table 4 ). In women treated specifically with immunossuppressants, the occurrence of obstetric and neonatal complications was also lower (as well as PTB, FGR, and LBW in specific), but not PPH ( Table 4 ). Regarding obstetric adverse outcomes (composite outcome), women treated with salicylates or costicosteroids had a higher rate of complications, as well as women with previous bowel surgery ( Table 4 ). Women treated with salicylates had a higher frequency of operative delivery (90%, n = 18 vs 68.2%, n = 15), as well as those medicated with corticosteroids or biologics (100%, n = 2 vs 80%, n = 32 and 90%, n = 9 vs 78.1%, n = 25, respectively). Neonatal adverse outcomes (composite outcome) were less frequent in women treated with salicylates (10%, n = 2 vs 13.6%, n = 3), thiopurines (6.3%, n = 1 vs 15.4%, n = 4), corticosteroids (0%, n = 0 vs 12.5%, n = 5), or biologics (10%, n = 1 vs 12.5%, n = 4), and also women with previous bowel surgery (0%, n = 0 vs 14.3%, n = 5) ( Table 4 ). Regarding PPH, women treated with salicylates, thiopurines, or biologics had a higher rate of complications, as well as women with previous bowel surgery ( Table 4 ).

**Table 4:** Inflammatory bowel disease treatment and obstetric/neonatal/puerperal adverse outcomes

IBD treatment	Obstetric adverse outcomes (composite outcome) ^a)^	Preterm birth	Fetal growth restriction	Operative delivery ^b)^	Cesarean section	Neonatal adverse outcomes (composite outcome) ^c)^	Low birth weight	Postpartum hemorrhage
**Medicated IBD**								
Yes (n = 36)	52.8% (19)	8.3% (3)	13.9% (5)	80.6% (30)	41.7% (15)	8.3% (3)	8.3% (3)	19.4% (7)
No (n = 6)	66.7% (4)	16.7% (1)	0% (0)	83.3% (5)	50% (3)	33.3% (2)	16.7% (1)	0% (0)
**Immunossuppressants**								
Yes (n = 19)	42.1% (8)	5.3% (1)	10.5% (2)	78.9% (15)	47.4% (9)	5.3% (1)	0% (0)	26.3% (5)
No (n = 23)	65.2% (15)	13% (3)	13% (3)	78.3% (18)	39.1% (9)	17.4% (4)	17.4% (4)	8.7% (2)
**Salicylates**								
Yes (n = 20)	75% (15)	10% (2)	25% (5)	90% (18)	35% (7)	10% (2)	10% (2)	25% (5)
No (n = 22)	40.9% (9)	9.1% (2)	0% (0)	68.2% (15)	50% (11)	13.6% (3)	9.1% (2)	9.1% (2)
**Thiopurines**								
Yes (n = 16)	50% (8)	6.3% (1)	12.5% (2)	75% (12)	37.5% (6)	6.3% (1)	0% (0)	25% (4)
No (n = 26)	50% (13)	11.5% (3)	11.5% (3)	84.6% (22)	46.2% (12)	15.4% (4)	15.4% (4)	11.5% (3)
**Corticosteroids**								
Yes (n = 2)	100% (2)	0% (0)	0% (0)	100% (2)	50% (1)	0% (0)	0% (0)	0% (0)
No (n = 40)	52.5% (21)	10% (4)	12.5% (5)	80% (32)	42.5% (17)	12.5% (5)	10% (4)	17.5% (7)
**Biologics**								
Yes (n = 10)	50% (5)	10% (1)	0% (0)	90% (9)	60% (6)	10% (1)	0% (0)	30% (3)
No (n = 32)	59.4% (19)	9.4% (3)	15.5% (5)	78.1% (25)	37.5% (12)	12.5% (4)	12.5% (4)	12.5% (4)
**Previous bowel surgery**								
Yes (n = 12)	58.3% (7)	8.3% (1)	16.7% (2)	75% (9)	50% (6)	0% (0)	0% (0)	16.7% (2)
No (n = 35)	54.3% (19)	8.6% (3)	8.6% (3)	74.3% (26)	37.1% (13)	14.3% (5)	11.4% (4)	14.3% (5)

Abbreviation: IBD, inflammatory bowel disease.

a) Obstetric adverse outcomes (composite outcome) included preterm birth, vaginal bleeding in the 3 ^rd^ trimester, fetal growth restriction, spontaneous and therapeutic abortion, stillbirth, placental abruption, premature rupture of membranes at term and preterm, prolonged premature rupture of membranes, preeclampsia, eclampsia, chorioamnionitis, congenital anomalies, intrahepatic cholestasis of pregnancy, anemia, oligohydramnios, short cervix, and gestational diabetes. b) Operative delivery included vaccum delivery, forceps delivery, and all cesarean sections. c) Neonatal adverse outcomes (composite outcome) included low birth weight, Apgar index < 7, neonatal death, and neonatal intensive care unit admission

Newborns of women with UC had a higher rate of LBW, neonatal death, and neonatal intensive care unit admission than women with CD but not Apgar index < 7 ( Table 5 ).

**Table 5:** Neonatal adverse outcomes

	IBD (n = 48)	CD (n = 28)	UC (n = 20)
LBW	10.4% (5)	3.6% (1)	20% (4)
Apgar index < 7	2.1% (1)	3.6% (1)	0% (0)
Neonatal intensive care unit admission	2.1% (1)	0% (0)	5% (1)
Neonatal death	2.1% (1)	0% (0)	5% (1)

Abbreviations: CD, Crohn's disease; IBD, inflammatory bowel disease; LBW, low birth weight; UC, ulcerative colitis.

## Discussion

In the cohort study carried out by Uma Mahadevan's team, adverse pregnancy outcomes/complications (including PTB, small for gestational age, stillbirth, placental abruption, preeclampsia and PROM, among other outcomes) were more frequent in pregnant women with IBD than in healthy pregnant women. 2

Most studies suggest IBD can contribute to increased frequency of PTB, although they do not specify the degree of disease activity or the therapy used. 4 5 6 7 8 9 12 20 21 26 27 28 29 In our study, PTB rate was 10.5% in women with UC, which is higher than the prevalence found in healthy populations according to most works (4.5–9.5%). 2 7 12 22 26 28 29 However, the frequency of PTB found in women with IBD in general (8.5%) or women with CD (7.1%) was similar to the refered baseline prevalence in the population. 2 7 12 22 26 28 29 Stephansson et al. 28 showed higher occurrence of PTB and small size for gestational age in women with IBD in a population-based prevalence study. However, Bortoli et al. 7 conducted a prospective case-control study that did not show significant differences regarding pregnancy outcomes such as spontaneous and therapeutic abortions, infant death *in utero* , and PTB between pregnant women with or without IBD. It is important to mention that in Bortoli's study and in the present article most pregnant women with IBD were in remission in the periconceptional period and remained with inactive disease during pregnancy.

Spontaneous and/or therapeutic abortions are also another of the outcomes generally studied in pregnant women with IBD, as was the case of the work carried out by Boyd et al., 27 without differences between the groups. 27 In our IBD population, there was 1 selective feticide in a woman with CD, corresponding to 2.1% of IBD women and 3.6% of CD, which is a higher rate than the rate of therapeutic abortion reported by Bortoli et al. 7 in a population without IBD (0.5–1.4%).

The possible association of IBD with congenital anomalies is controversial, despite having been addressed in several publications; in the IBD population included in our work there where no cases of congenital anomalies, whereas the prevalence in the general population is higher and disparate in the literature (0.7–7%). 2 4 7 9 12 22 27 28 29 30 Regarding other pregnancy complications included in our study, preeclampsia rates (4.3% in all IBD types, 3.6% if CD and 5.3% if UC) were similar to those reported in healthy populations in the literature (1.8–5.6%). 2 9 12 27 28 31 The placental abruption rates of our IBD population were higher than the ones relative to the general population (2.1% vs 0.4-1.2%), just like those of gestational diabetes (8.5% vs 1.9-5.8%) and PROM (21.2% vs 2.8%), specialy in women with CD (14.3% vs 1.9% regarding gestational diabetes and 25% vs 2.8% respecting PROM). 2 9 26 31

The literature suggests a higher frequency of cesarean sections in pregnant women with IBD compared to healthy ones. 4 5 10 22 26 28 This is also supported by our results: 39.6% of women diagnosed with IBD (50% of those with CD and 25% of women with UC) had cesarean sections, whereas the frequency of c-section in the general population is 9.5–28.2%, according to the literature. 2 7 12 22 26 31 According to our work, operative delivery was more frequent in pregnant women with IBD than in the population without IBD of the Sultan's team study (72.9% vs 35.6%), specially if IBD was active in the periconceptional period (100% vs 35.6%). 31 As expected, all pregnant women with perianal disease had cesarean section, as this is an indication for this according to the European Crohn's and Colitis organization's (ECCO) guidelines. Cesarean delivery was also more frequent if IBD had been diagnosed > 5 years ago, which may be explained by the cumulative complications of the disease.

In the literature, there are few studies that evaluated puerperal outcomes, and, in those that did, PPH was the most studied, and the results were mixed. 26 31 In the IBD population of our study, the rate of PPH was 14.9%, higher than the prevalence described by the work of Sultan et al. 31 —8.7%.

The literature is not consensual regarding adverse neonatal outcomes. Some studies observed a higher frequency of LBW in children of mothers with IBD. 4 20 29 These results were supported by our work: newborns of women with IBD, and speciffically UC, had higher rates of LBW (10.4% and 20%, respectively) than the general population (4.5–10%), unlike newborns of women with CD (3.6%). 2 29 31 In one publication, UC was associated with neonatal death, and in our IBD population, the prevalence of neonatal death in IBD women, specially if UC (2,1% and 5%, respectively), was higher than the rate reported in the literature for women without IBD (0.2%). 2 22 In our work, the rate of Apgar index < 7 in newborns of women with IBD was higher than that reported for healthy population in the literature (2.1% vs 0.7–1.4%). 22 27 28 However, other studies did not find differences between the groups regarding adverse neonatal outcomes, either as a composite or isolated outcome. 2 5 6 7 8 9 26

Most publications suggest that disease activity is associated with adverse obstetric outcomes, like in our study, regarding IBD activity in the periconceptional period (but not during pregnancy). 32 33 34 However, in the prospective cohort study by Lima-Karagiannis et al., 35 the activity of IBD did not confer a greater risk of obstetric or neonatal complications. In our study, women with active IBD (in the periconceptional period and during pregnancy) had more operative delivery and neonatal adverse outcomes (as a composite outcome and LBW in especific) but not higher rate of PPH.

Inflammatory bowel disease severity and its potential association with obstetric/neonatal/puerperal complications is generally not addressed in publications in this area. In our work, women with more severe CD had higher rates of neonatal adverse outcomes and PPH but not obstetric complications; UC severity did not seem to relate to poor outcomes. However, it is important to state that IBD severity was the parameter with more missing information, and this may lead to more unreliable results.

Women with diagnosis of IBD > 5 years prior had more obstetric and neonatal complications, assessed as composite outcomes (including PTB and LBW individually), and also more PPH. To justify these results, we hypothesize that women with a longer time since diagnosis are more likely to accumulate complications of the disease than women with a more recent diagnosis.

Women with medicated IBD had less obstetric/neonatal complications (composite outcomes), including PTB and LBW individually, probably because the treatment leads to better control of the disease symptoms, activity, and/or severity.

Women treated with salicylates had a higher frequency of operative delivery (90% vs 68.2%), and this may be explained by the fact that salicylates are a non-steroidal antiinflammatory substance, and it inhibits the synthesis of prostaglandins, which may contribute to the inhibition of labor and its prolonged duration, leading to a higher incidence of operative delivery.

In this study's population, some pregnant women with IBD had medical comorbidities theoretically associated with a higher risk of PTB, LBW, and cesarean section, which could be a confounding factor in the interpretation of the results. One of the biases of this work, which comes from its retrospective nature, was the impossibility of collecting information on the degree of activity, severity, duration and therapy of IBD, as well as the presence/absence of perianal disease for all pregnant women with IBD. This aspect allied to the heterogenity of the IBD group may have contributed to possibly inaccurate and sometimes disparate results from the available literature.

However, we consider that the analysis of these particularities of the IBD constituted a strong point of this work, as they are generally not addressed in publications in this area. Also, the fact that the sample refers to a tertiary center offers a different perspective from that usually portrayed in the literature—even more heterogeneous samples often coming from national databases, pregnant women less medicated and rarely undergoing biological therapy (either because of the length of the studies or because the severity of the disease does not justify it). 26 This disparity between the studies, not only in terms of methodology but also in sampling, may explain the differences in results.

All in all, it is important to mention that all the results should be considered in a very specific context and their interpretation is limited, not only because of all the factors mentioned before but also because the sample was relatively small and heterogenous.

## Conclusion

The ocurrence of some obstetric complications (placental abruption, gestational diabetes, and PROM) was higher in the IBD population (and specialy if CD) in this work than the prevalence described in the literature for healthy individuals, and the same tendency was verified with PTB but only in UC cases. 2 7 9 12 22 26 28 29 31 Other adverse obstetric outcomes in women with IBD had similar prevalences to the ones described in the literature for healthy pregnant women (PTB and preeclampsia), or even lower (congenital anomalies). 2 4 7 9 12 22 26 27 28 29 31 Regarding cesarean sections, the rate in our IBD population was higher than the reported in the literature for the general population, specialy if CD. 2 7 12 22 26 31 The same tendency occurred with PPH and some neonatal adverse outcomes (LBW, Apgar index < 7 and neonatal death). 2 22 27 28 29 31

Obstetric and neonatal complications (assessed as composite outcomes) were more frequent in women with UC than with CD but the opposite tendency was verified regarding PPH. Cesarean sections in the IBD population were more frequent if CD, active IBD in the periconceptional period, diagnosis of IBD > 5 years ago or active perianal disease. Women with active IBD (in the periconceptional period or during pregnancy) had more neonatal adverse outcomes (as a composite outcome and LBW individually) but not higher rate of PPH. Diagnosis of IBD >5 years prior may be associated to more obstetric and neonatal complications, assessed as composite outcomes, including PTB and LBW in particular. Women with more severe CD had higher rates of neonatal adverse outcomes (composite outcome) and PPH but not obstetric complications (composite outcome). Ulcerative colitis severity did not seem to relate to poor outcomes. Women with medicated IBD had less obstetric and neonatal complications (composite outcomes), including PTB and LBW individually, probably because the treatment lead to better control of the disease symptoms, activity, and/or severity. This work reinforces the importance of therapeutic optimization and control of IBD prior to pregnancy and adequate gastroenterological surveillance during pregnancy. We also highlight the importance of obstetric and postpartum vigilance in this population, allowing early diagnosis and treatment of potential complications. However, specially due to the retrospective nature of this study and to a small and heterogenous sample, these results should be interpreted carefully.
